# THE IMPACT OF THE USE OF SYMBIOTICS IN THE PROGRESSION OF NONALCOHOLIC FATTY LIVER DISEASE IN A RAT MODEL

**DOI:** 10.1590/0102-6720201700030011

**Published:** 2017

**Authors:** Eliane TAGLIARI, Antonio Carlos CAMPOS, Thais Andrade COSTA-CASAGRANDE, Paolo Rogério SALVALAGGIO

**Affiliations:** 1 Department of Industrial Biotechnology Graduate, Positivo University, Curitiba, PR;; 2 Department of Surgery, Federal University of Paraná, Curitiba, PR, Brazil

**Keywords:** Non-alcoholic fatty liver disease, Probiotics, Microbiota

## Abstract

**Background:**

Non-alcoholic fatty liver disease (NAFLD) is characterized by accumulation of intrahepatic lipid. The use of live microorganisms promotes beneficial effects; however, the use of symbiotic and its role in NAFLD is not yet fully understood.

**Aim:**

Verify if the symbiotic administration influences the occurrence and progression of NAFLD in rats, after induction of hepatic steatosis by high calorie diet.

**Method:**

Forty-five rats were divided into four groups: G1 (control); G2 (control+symbiotic); G3 (high calorie+symbiotic) and G4 (high calorie), and euthanized after 60 days of diet. Liver disease was evaluated by biochemical analysis, IL6 measurement and histological assessment.

**Results:**

Symbiotic had influence neither on weight gain, nor on coefficient dietary intake in G3 and G4. G2 had the greatest weight gain, while G1 had the highest coefficient dietary intake between groups. G1 showed higher expression of aspartate aminotransferase than those from G2 (150±35 mg/dl, and 75±5 mg/dl) while G4 showed higher expression of the enzyme compared to G3 (141±9.7 mg/dl to 78±4 mg/dl). Liver histology showed different stages of NAFLD between groups. G4 animals showed increased serum interleukin-6 when compared to G3 (240.58±53.68 mg/dl and 104.0±15.31 mg/dl).

**Conclusion:**

Symbiotic can reduce hepatic aminotransferases and interleukin-6 expression. However, the histology showed that the symbiotic was not able to prevent the severity of NAFLD in rats.

## RESUMO

**Racional:**

A doença hepática gordurosa não alcoólica (DHGNA) é caracterizada por acúmulo de lipídios intra-hepáticos. O uso de microrganismos vivos promove diversos efeitos benéficos; porém, a utilização de simbióticos e sua atuação na DHGNA ainda não está totalmente esclarecida.

**Objetivo:**

Verificar se a administração de simbióticos influencia na ocorrência e na progressão da DHGNA em ratos, após a indução de esteatose hepática por dieta hipercalórica.

**Método:**

Quarenta e cinco ratos Wistar foram divididos em quatro grupos: G1 (controle); G2 (controle+simbiótico); G3 (hipercalórica+ simbiótico) e G4 (hipercalórica), e eutanasiados após 60 dias de dieta. Coleta de sangue foi realizada para obtenção de análises bioquímicas e dosagem de IL6, e de tecido para análise histológica do fígado**.**

**Resultados:**

O simbiótico não influenciou no ganho de peso e no coeficiente de consumo alimentar nos grupos G3 e G4. Já G2 obteve maior ganho de peso, enquanto G1 apresentou o maior coeficiente de consumo alimentar entre os grupos. G1 apresentou maior expressão de aspartato aminotransferase em relação ao G2 (150±35 mg/dl e 75±5 mg/dl), enquanto G4 teve maior expressão desta enzima em relação ao G3 (141±9,7 mg/dl e 78±4 mg/dl,). A análise histológica hepática mostrou diferentes estágios de evolução da DHGNA entre os grupos. Animais do G4 apresentaram aumento sérico de interleucina-6 quando comparados a G3 (240,58±53,68 mg/dl, e 104,0±15,31 mg/dl).

**Conclusão:**

Os simbióticos reduziram aminotransferases hepáticas e a expressão de interleucina-6. No entanto, a histopatologia demonstrou que o simbiótico não foi capaz de prevenir a severidade da DHGNA em ratos.

## INTRODUCTION

Non-alcoholic fatty liver disease (NAFLD) is characterized by accumulation of lipids in hepatocytes, which accounts for at least 5% of the weight of this tissue. It includes simple steatosis, non-alcoholic steatohepatitis (NASH), fibrosis, cirrhosis, and hepatocellular carcinoma. Population studies indicate that 10-50% of the population may have relatively benign hepatic steatosis. About 40% of patients with NAFLD develop into non-alcoholic hepatic steatosis, and 5% develop fibrosis after 4-13 years^3^.

It is believed that the development of DHGNA involves two phases. In the first one occurs the inadequate storage of lipids or ectopic accumulation of this nutrient. The second is marked by oxidative stress that causes hepatocyte injury and leads to the development of inflammation^29^.

When normal physiology of the liver is altered, and inflammatory cells are activated, changes in the mechanisms of control and the activation of factors derived from the intestine occur. The imbalance in the intestinal microbiota can generate lipopolysaccharides that are components of the cell wall of gram-negative bacteria that reach the liver through the portal vein, leading to endotoxemia^14^.

The production of endotoxin by the intestinal microbiota can cause inflammation in the liver of patients with obesity, diabetes, metabolic disease, hepatic steatosis and NASH^28^.

Studies using live microorganisms (probiotics) or soluble (prebiotic) fibers in the treatment of various diseases, including NAFLD, are well established in the literature^15^ ^,^ ^13^. However, the use of symbiotics (association of probiotics with prebiotics) in the control of NAFLD and the mechanisms by which they decrease the inflammatory process and the progression to the more advanced stages of NAFLD are still not fully understood.

The objective of the present study was to verify if the administration of symbiotics influences the occurrence of DHGNA in rats and to verify if the symbiotic conditions reduce the levels of liver enzymes and pro-inflammatory markers after induction of hepatic steatosis by hypercaloric diet.

## METHODS

### Animals

The animal experiments were carried out under institutional terms according to the norms established in Federal Law No. 11,794, of October 8, 2008, with the norms established by the National Council for the Control of Animal Experimentation (CONCEA) and after approval by the Ethics Committee Of Animals (CEUA) from Universidade Positivo, protocol number 181-2013.

A total of 45 adult male Wistar rats weighing approximately 200 g were collected from the Positivo University laboratory. The animals were kept in controlled environment conditions, with temperature, luminosity and humidity control of the air. They were divided into four groups as symbiotic supply and diet provided.

### Diet and symbiotic

Before the experiment itself, a pilot study of steatosis induction was carried out in five rats over a period of 30 days, according to the methodology used by Cintra et al.^9^. The animals were induced to the hypercaloric diet (association of hyperlipidemic+hyperlipid diet) in order to represent a more realistic profile of the western diet rich in carbohydrates and fats^2^ ^,^ ^18^ ^,^ ^23^ ^,^ ^27^. After confirming the standard model to be followed during the pilot study, the experiment was performed by dividing the animals into four groups of 10 animals each, namely: Group 1 (G1) - with standard feed indicated by the vivarium for 60 days; Group 2 (G2) - with standard diet plus symbiotic for 60 days; Group 3 (G3) - with a hypercaloric diet for 30 days to induce steatosis and after this period the hypercaloric diet was maintained plus symbiotic for another 30 days, totaling 60 days; Group 4 (G4) - with a hypercaloric diet for 30 days to induce steatosis, maintaining it for another 30 days, totaling 60 days. 

**FIGURE 1:**
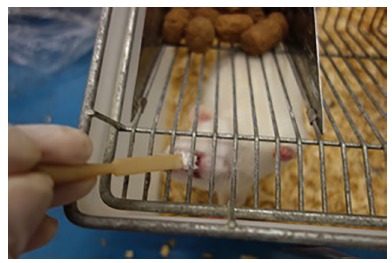
Oral administration of the symbiotic

The distribution of the diets and the symbiotic during the experiment was thus determined: standard diet of the vivarium: Presence® Labina diet; hyperglycemic diet: 20% water with fructose ad libidum (in which 200 g of pure fructose was added to each liter of water); hyperlipid diet developed according to studies by Cintra et al^9^ and Reeves et al^24^ in the following proportion: diet composed of standard diet of AIN93-G (American Institute Nutrition, AIN-1993) + 35% animal fat, developed by PRAG Soluções Ltda. *Lactobacillus paracasei* LPC-37, *Bifidobacterium lactis* HN0019, *Lactobacillus rhamnosus* HN001, *Lactobacillus acidophilus* NCFM^®^and fructooligosaccharide (5.5 g) at the daily dose of 200 mg/kg/day were used as the symbiotic agent with strains (doses 1x109 UFC/g) day/animal, orally, corresponding to the approximate dose of 200,000 to 206,109 CFU (colony forming units) administered seven times a week for 30 days orally with the aid of a spatula adapted for this research (Figure 1 ). This dosage was chosen because it corresponds, at least in part, to the daily dosage indicated for humans (about 14 g)

### Animal and laboratory procedures

During the experiment, we performed a weighing of the remaining diet every two days. The animals’ body weight was recorded every 30 days to evaluate the weight gain. These data were used to determine the coefficient of food efficiency (CEA)^5^. It was calculated using the equation: CEA=[weight gain (g)]/[food consumption (g)]. After 60 days the animals were anesthetized with inhalation of isoflurane to collect 4 ml of blood (cardiac puncture) per animal immediately before the euthanasia procedure.

Serum levels of triglycerides, total cholesterol and aspartate aminotransferase (AST or TGO) and alanine aminotransferase (ALT or TGP) levels were evaluated at the end of the experiment. The amount of IL-6 in the blood was also dosed with a kit (IMMU-EK0412-IL-6 Rat Elisa Kit, Boster Biological, Fremont, CA, USA) at the end of the experiment. The animals were euthanized with isoflurane inhalation followed by carbon dioxide intoxication in a CO2 chamber (Baxter®, Chicago, USA). Afterwards, the liver and intestine were collected and placed in formaldehyde 10% for fixation and after 48 h they were embedded in paraffin, cut into a microtome, 5 μm thick, stained with H&E and analyzed for the development of steatosis in histopathology. Histological analyzes of the intestine were performed in order to evaluate changes in the architecture, lymphoid follicle and the presence or absence of acute or chronic inflammation. In the liver, the degree of inflammation and its intensity (mild, moderate or severe) and the percentage of fat cells were measured, and the classification system was adapted from Brunt et al.^5^.

### Statistical analysis

Quantitative variables were described in mean and standard error. For the comparison of the groups, the variance analysis model was considered. When, at some point, the hypothesis of equality of the averages of the four groups was rejected, they were compared two by two, considering the LSD test. For the comparisons of each evaluation moment with the first evaluated moment the Student t test for paired samples was considered. For the evaluation of the interaction between evaluation time and group, for the feed intake variable, the Split-Plot model was considered. The non-parametric Kruskal-Wallis test was used to compare the groups in relation to the steatosis classification. For the evaluation of the normality condition of the variables, the Jarque-Bera test was considered. P values less than 0.05 indicated statistical significance.

## RESULTS

All groups presented weight gain at the end of the experiment and Table 1 shows their evolution. The G1 group, with control diet, presented lower final weight gain in relation to the other groups. The G1 and G2 groups presented lower final weight gain in relation to the groups with a G3 and G4 hyperlipid diet. When the groups that used the symbiotic group (G2xG3) were compared, the control group had lower gain at 30 days (292.4x343.2, p <0.001). When compared the group that used the control diet to the group that used symbiotic (G1xG2), G2 had greater weight gain (p <0.001). When comparing the groups that used the hypercaloric diet (G3xG4), the use of the symbiotic (G3) did not influence the weight gain (p> 0.05).

**TABLE 1:** Animals weight evaluation

Group	Initial weight	30 days	60 days
G1	241.4±17.2	326.4±13.1	363±20.7
G2	259.5±20.8	292.4±28.1	396.8±43.6
G3	255.8±20.1	343.2±35.3	425.6±33.8
G4	260.3±17.4	337±24.1	433.4±23

Feed, water and fructose consumption were evaluated and all groups presented similar consumption in the first weeks. After the third week, the G3 and G4 feed consumption decreased by 15% in relation to the other groups. The consumption of water and fructose remained similar throughout all time in all groups.

**FIGURE 2:**
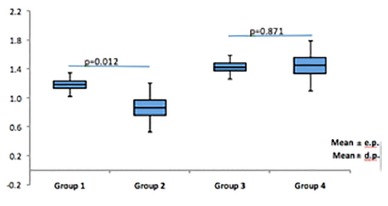
Coefficient of food efficiency - CEA - of animals

When comparing the groups with control diet (G1 and G2) the G1 group had higher CEA (p=0.012). When comparing the groups with hypercaloric diet consumption was similar between groups, G3=G4 (Figure 2).

**FIGURE 3:**
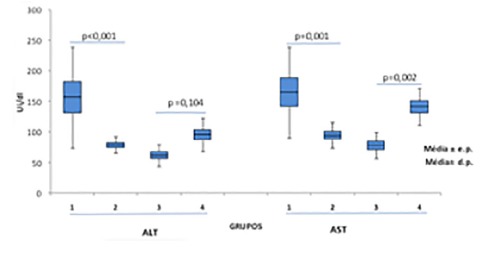
Determination of aminotransferases (groups)

Figure 3 shows the values of the aminotransferases obtained at the end of the experiment. The control group (G1) had the highest expression of ALT (150±35 mg/ml in G1 vs. 75±5 mg/ml in G2, p <0.001). When comparing the groups with hypercaloric diet, the group (G4) had higher ALT expression (62.1±5 mg/ml in G3 vs.95.6±8.6 mg/ml in G4, p=0.104). Also in Figure 3 compared to AST, animals from the control group (G1) had AST increase (165±23 mg/ml in G1 vs. 94±6 mg/ml in G2, p=0.001). When comparing the groups with hypercaloric diet, the group (G4) had higher AST expression (78±4 mg/ml in G3 vs.141±9.7 mg/ml in G4, p<0.002). The groups that used the symbiotic (G2 and G3) had a smaller variation of AST.

Cholesterol values did not differ between groups and did not change from reference values for the species^16^. The control group (G1) had a higher triglyceride value than the G3 group (102.9 mg/dl vs. 61.5 mg/dl, p <0.001), but none of the groups had higher values than the reference species group^16^.

Table 2 expresses the histological analysis of the degree of steatosis between the groups. The groups supplemented with the symbiotic G2 and G3 presented steatosis, but the evolution of the disease was different between them. In group G2 20% of the animals did not develop the disease, and mild steatosis predominated (60%); already in G3 all developed disease, predominating moderate steatosis (50%), followed by mild (40%). Both groups had a proportion of 10% of the animals that progressed to severe steatosis.

**TABLE 2:** Histological evaluation of animals liver

Group	Steatosis								Total	p
	0 (absent)		1 (mild)		2 (moderate)		3 (severe)			
	n	%	n	%	n	%	n	%		
1	4	40%	4	40%	2	20%	0	0%	10	
2	2	20%	6	60%	1	10%	1	10%	10	
3	0	0%	4	40%	5	50%	1	10%	10	0,030
4	0	0%	4	40%	6	60%	0	0%	10	

When G1 and G4 were compared, in G1 40% of the animals did not present steatosis, 40% had mild and 20% moderate steatosis. In G4, moderate (60%) and mild (40%) steatosis predominated. Both groups did not show a similar course for severe steatosis.

**FIGURE 4:**
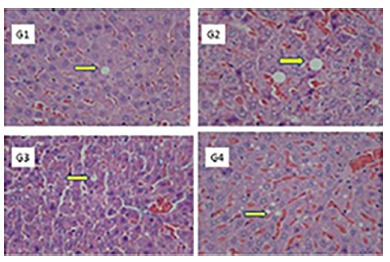
Hepatic histological sections (100x) with absence of inflammation and fibrosis and steatosis (arrow)

Figure 4 shows the histopathological aspects with steatosis between the groups. The groups that received the control diet (G1 and G2) presented a lower degree of steatosis evolution, when compared to the other groups (G3xG4), who received the hypercaloric diet. Among the groups that received the symbiotic (G2xG3), the G2 group had a lower steatosis evolution.

All animals of all groups presented preserved cellular architecture and without chronic or acute inflammation in the intestine. In the G2 and G4 groups, 10% of the animals presented alterations in the lymphoid follicle; in G3, 20%.

**FIGURE 5:**
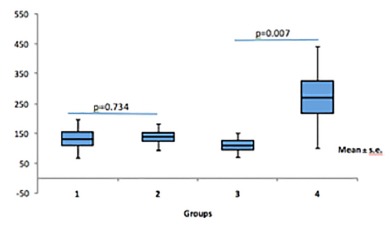
Evaluation of IL-6 in groups

Figure 5 expresses the assessment of IL-6 between the study groups. The animals in the G1 (105.66±23.32 mg/dl) and G2 (124.90±13.80 mg/dl) groups did not present statistical differences (p=0.734) regarding serum IL-6 expression. When G3 (104.0±15.31 mg/dl, p<0.028) x G4 (240.58±53.68 mg/dl, p<0.007) was compared, G4 had higher serum IL-6 expression. Among the groups that used the symbiotic G2 (124.0±13.80 m /dl) vs. G3 (104.0±15.31 mg/dl) there was no statistical difference regarding IL-6 expression, p<0.389.

## DISCUSSION

The role of the symbiotic in controlling the progression of hepatic steatosis is not fully understood. The central objective of this research, after eight weeks of experimental protocol, was to evaluate if the consumption of a symbiotic can impact the development of NAFLD in rats.

It was observed that the symbiotic reduced levels of aminotransferases but was unable to alter the histological progression of NAFLD in an experimental model used. The difference in steatosis evolution may be related to diet composition. However, steatosis does not normally progress to the advanced stage of fibrosis in experimental models^5^. The results may also be related to the intestinal microbiota, since the metabolic profile and bacterial composition of the microbiota determine a greater or lesser inflammatory response, depending on the predominant host strains^19^. Symbiotics may also alter the synthesis of short chain fatty acids (SCFA) due to the presence of *Bifidobacterium*, *Lactobacillus* and oligosaccharides^6^ ^,^ ^26^. SCFA can alter the expression of genes involved in neoglycogenesis or beta-oxidation, thereby increasing the energy extraction from diet and intrahepatic fat. SCFA may also alter appetite signaling and expression of genes involved in the inflammatory response^11^.

The most important finding of this study was the reduction of IL-6 with the use of symbiotics. IL-6 is involved not only in the response of acute liver injury, but also in adipose tissue metabolism, lipoprotein lipase activity, and hepatic triglyceride secretion^12^. These results show that the animals that received a hypercaloric diet had higher expression of IL-6 when compared to the control groups, which received similar diet, but supplemented with symbiotics. Feldstein et al^12^ demonstrated greater expression of IL-6 in the liver of patients with NAFLD, thus correlating it positively with the severity of inflammation and fibrosis.

Other human studies reported similar results to the present study. Probiotic therapies reduced hepatic transaminases, TNF-a and IL-6 expression and improved insulin resistance in patients with DGNHA^10^ ^,^ ^19^. Recent results have shown that symbiotics increase levels of *Bifidobacteria* and *Lactobacillus* in the gut of patients with colon cancer and irritable bowel syndrome. Future studies should specifically investigate the role of specific bacteria in NAFLD^25^.

Other conclusions of this study deserve special attention. The first is related to the experimental model. Induction of steatosis in rodents usually uses a hyperlipidic or hyperglycemic diet, under sedation and through a feeding tube, which goes from 21 to 30 days^1^ ^,^ ^4^ ^,^ ^16^ ^,^ ^30^. We chose a hyperlipidic and hyperglycemic diet combined, which represents a more realistic profile of the western diet. A spatula was used for symbiotic administration^23^. No previous report of this symbiotic delivery method has been found to date. This simple, reproducible and innovative method of symbiotic delivery with a spatula without the need for a feeding tube or sedation may certainly contribute to scientific research, allowing in future studies other strains and dosages of bacteria can be used in the same way. Second, the symbiotic was unable to control weight gain in this study. Similar results were found by Assis et al.^2^. More studies are needed to determine whether the symbiotic can alter body fat deposition and weight gain. Third, it is believed that symbiotic consumption could explain the lowest level of CEA in G2. The possible mechanisms that involve the loss of body weight may have occurred through the stimulation of incretins, such as glucagonlike peptide-1 (GLP1) and neuropeptide-Y (NPY), triggering satiety and consequently reducing food consumption^7, 22^.

Finally, the symbiotic had no impact on cholesterol levels. A recent meta-analysis has shown controversial results of cholesterol reduction with the use of symbiotics^7^. In addition, the reduction of triglycerides with the use of symbiotics was not statistically significant in this study, similar to the findings of Cani et al.^8^.

The present study had some limitations. The first is the short period of follow-up of the animals. The second refers to the fact that we chose to evaluate a single inflammatory marker (IL-6). Finally, common bacteria strains were used in dosage that mimics human use. It is possible that different combinations of different strains and dosages, as well as the composition of the intestinal microbiota of the animals, may interfere in the results. In the future, as metagenomic advances progress, more precise knowledge of all the mechanisms involved in the gut-liver relationship may possibly be predicted, possibly predicting the severity of NAFLD.

In summary, administration of symbiotics was associated with reduced levels of aminotransferases and IL-6 expression. However, the symbiotic was unable to prevent the progression of hepatic steatosis in rats.

Further studies should be performed, expanding the use of other markers and confirming the chosen model of oral symbiotic administration.

## CONCLUSION

Symbiotic can be an alternative in the treatment of NAFLD, because according to the results found it was able to reduce hepatic aminotransferases and to reduce the expression of IL6. However, contrary to the biochemical analysis, histology showed that the symbiotic was not able to prevent the severity of the disease.
